# A new inverse regression model applied to radiation biodosimetry

**DOI:** 10.1098/rspa.2014.0588

**Published:** 2015-02-08

**Authors:** Manuel Higueras, Pedro Puig, Elizabeth A. Ainsbury, Kai Rothkamm

**Affiliations:** 1 Centre for Radiation, Chemical and Environmental Hazards, Public Health England, Chilton, Oxfordshire OX11 0RQ, UK; 2 Departament de Matemàtiques, Universitat Autònoma de Barcelona, Bellaterra, Barcelona 08193, Spain

**Keywords:** Bayesian calibration, biological dosimetry, radiotherapy, calibrative density, compound Poisson distribution, Hermite distribution

## Abstract

Biological dosimetry based on chromosome aberration scoring in peripheral blood lymphocytes enables timely assessment of the ionizing radiation dose absorbed by an individual. Here, new Bayesian-type count data inverse regression methods are introduced for situations where responses are Poisson or two-parameter compound Poisson distributed. Our Poisson models are calculated in a closed form, by means of Hermite and negative binomial (NB) distributions. For compound Poisson responses, complete and simplified models are provided. The simplified models are also expressible in a closed form and involve the use of compound Hermite and compound NB distributions. Three examples of applications are given that demonstrate the usefulness of these methodologies in cytogenetic radiation biodosimetry and in radiotherapy. We provide R and SAS codes which reproduce these examples.

## 1. Introduction

In spite of strict safety measures and regulations, radiation accidents or unplanned exposures occur, for instance in radiology services and radiotherapy departments at hospitals, or using radiography cameras in industry. There have also been some major radiation/ nuclear accidents, such as Chernobyl or Fukushima, that have affected many people [1]. In the event of a radiation accident, biological dosimetry is essential for the timely determination of the radiation dose to which an individual has been exposed. On the other hand, radiotherapy is commonly used to treat cancerous tumours, and it is important to know the total absorbed blood dose to prevent possible complications or side effects. Biological dosimetry relies on quantifying the amount of damage induced by radiation at a cellular level, for instance by counting dicentrics or micronuclei. These aberrations appear because when cells are exposed to radiation, breaks are induced in the chromosomal DNA and the broken fragments may rejoin incorrectly. Therefore, the frequency of chromosome aberrations increases with the amount of radiation and is a reliable and very well-established biological indicator of radiation absorbed dose. Such information supports the clinical management of a patient, enables rapid triage in the case of a large-scale radiation incident and reassures the ‘worried well’ that they have not received a severe radiation dose. At high acute whole body doses above 2 Gy, haematopoietic failure (or myelodysplasia) is the primary threat associated with acute radiation syndrome which can be supported by early treatment with cytokines or, at very high doses, bone marrow transplants [2]. To estimate the dose absorbed by an individual, dose–effect calibration curves are required which are produced by irradiating peripheral blood lymphocytes to a range of doses. The protocol and methodology for such calibration experiments is described in a recent manual of the International Atomic Energy Agency [3].

The usual approach for constructing the calibration curve is to irradiate *n* blood samples from various healthy donor with several doses *x*_*i*_, *i*=1,…,*n*. Then, for each irradiated sample, *n*_*i*_ cells are examined and the numbers of observed chromosomal aberrations *y*_*ij*_, *j*=1,…,*n*_*i*_ is recorded. For the dicentric assay, it is usually assumed that the counts *y*_*ij*_ follow a Poisson distribution [4] or a compound Poisson distribution [5] whose mean is a function of *x*_*i*_ and a set of parameters *β*, i.e. *E*(*y*_*ij*_)=*f*(*x*_*i*_,*β*). From the point of view of IAEA [3], *β* are the calibration coefficients and *f*(*x*_*i*_,*β*) is the mean of aberrations per cell (called yield or frequency of aberrations per cell, in the cytogenetics field). The parameters of this regression model are usually estimated by maximum likelihood [6], and the MLE and its estimated variance–covariance matrix are calculated and recorded. Therefore, in the case of an irradiated patient, a blood sample is taken and *m* lymphocytes are scored obtaining the counts ${\tilde{y}}_{1},\ldots ,{\tilde{y}}_{m}$. The classical approach to estimate the absorbed dose *x* and its confidence limits is to use the inverse regression method of Merkle [7], also described as a standard procedure in [3]. An improved classical inverse regression method applied to Electron Paramagnetic Resonance dosimetry is found in [8].

Bayesian approaches allow simple incorporation of prior information concerning the circumstances of the exposure. Groer & Pereira [9] were the first to investigate the use of Bayesian models in chromosome dosimetry, for neutron exposure, and since then several researchers have used Bayesian methods in radiation biodosimetry. For instance, Di Giorgio & Zaretzky [10] used a Bayesian approach to present the uncertainty on a biological dose estimate for a radiation overexposed patient in Latin America: a Poisson model with a Jeffrey's prior was used and it was further demonstrated that the Bayesian approach allows presentation of probabilities for dose ranges, which leads to a much more intuitive interpretation of the biological dosimetry results. A review of these methods can be found in [11]. There is also one recent program, CytoBayesJ [12], which provides some basic software tools for Bayesian analysis of cytogenetic radiation dosimetry data.

In this paper, we present a new Bayesian-type method to use cytogenetic data to estimate the dose to which a patient has been exposed. This method uses dose–effect calibration curves estimated by the classical (frequentist) approach suggested in the IAEA manual. Therefore, our new method has the advantage that allows reanalysis of many of the published examples of radiation exposures that were studied using the classical methods. In addition, the method is in fact a general inverse regression model for count responses that could also be applied in contexts other than radiation biodosimetry.

For the three routines implemented in the R statistical software (v. 3.1.1) run in the examples (§§3a,b and 4a) see the electronic supplementary material. A SAS (v. 9.3) routine for model (a) (see table 3) in §3a is also provided. A new R package called ‘radir’, which implements the Poisson response models presented here, is available under request to the corresponding author.

## 2. A Bayesian-type inverse regression model

The Poisson distribution is usually used to describe the distribution of dicentric chromosomes per cell when the patient has been irradiated with small doses and with a low linear energy transfer (low-LET radiation). However, after exposure to high-LET, acute radiation, the distribution of dicentrics per cell often presents overdispersion and therefore compound Poisson distributions are preferred. The commonly compound Poisson distributions in biodosimetry are the Neyman A (NA) [13], the negative binomial (NB) [14] and recently the family of *r*th-order univariate Hermite distributions [15]. These compound Poisson distributions, also known as stopped-Poisson distributions, can be justified by a simple physical model of chromosomal aberration formation: the particles traverse the cell nucleus following a Poisson process and, for each particle, there is a probability (the generalizing distribution) to produce *k* aberrations. Then the number of aberrations follows a compound Poisson distribution. In other words, a random variable *Y* follows a compound probability distribution if it can be represented by

2.1 $$Y=\sum_{i=1}^{N}\xi_{i},$$

where *N* is a count data random variable and *ξ*_1_,*ξ*_2_,… are independent, identically distributed random variables that are also independent of *N*. In the case where *N* is Poisson, *Y* is said to follow a compound Poisson distribution. The distribution of *ξ*_*i*_ is called the generalizing distribution. In particular when the distribution of *ξ*_*i*_ is Poisson, the distribution of *Y* is an NA, when *ξ*_*i*_ follows a logarithmic distribution, *Y* is NB distributed, and when *ξ*_*i*_ is distributed as a binomial with a number of trials equal to 2, then *Y* follows a Hermite distribution [16]. This can be expressed according to the Gurland's notation [16,17] as N⋁ξ. In particular, parametrizing with respect to the population mean *μ* and dispersion index *δ* (the ratio of the variance to the mean *σ*^2^/*μ*) we have the symbolic representation,

— NA(*μ*,*δ*)∼ Pois(μ/(δ−1))⋁ Pois(*δ*−1)
— NB(*μ*,*δ*)∼ Pois(μlog⁡(δ)/(δ−1))⋁ log((*δ*−1)/*δ*)
— Herm(*μ*,*δ*)∼ Pois(μ/2(δ−1))⋁ Bin(2,*δ*−1).

Properties, formulae and algorithms to calculate the probabilities of these distributions can be found in [16]. In brief, they are partially closed under addition [18], the maximum-likelihood estimator of the population mean is the sample mean and they are also members of the discrete exponential dispersion family of distributions. These properties are shared with other distributions potentially useful in biodosimetry, such as Polya Aeppli or Poisson-inverse Gaussian. See [18] for more properties and characterizations of these distributions. In particular, given a random variable *Y* (with mean *μ* and dispersion index *δ*) belonging to one of these models, the sum of *n* independent copies of *Y* also belongs to the same model having the same dispersion index and a mean equal to *nμ*. Moreover, if *δ* is known, the sum of the observations is a sufficient statistic for *μ*, containing all the information of the model. This is an important property that will be used in §4.

Let *D*={(*x*_*i*_,*y*_*ij*_)}, *i*=1,…,*n*, *j*=1,…,*n*_*i*_ be a calibration dataset where each *y*_*ij*_ represents a count data observation which will be assumed to follow a Poisson distribution or a two-parameter compound Poisson distribution. Here *x*_*i*_ are the values of the independent variable, dose in the case of cytogenetic radiation biodosimetry. The number of different exposed doses is *n* and *n*_*i*_ is the sample, the number of blood cells for the *i*th dose. For all the models, we define the regression function *E*(*y*_*ij*_)=*f*(*x*_*i*_,*β*), $\beta \in R^{p}$. Moreover, for compound Poisson modelling, we assume that the dispersion index is a constant (*δ*). In practice, this assumption could be verified by plotting the empirical values of the dispersion index ($s_{y_{i}}^{2}/{\bar{y}}_{i.}$) against the *x*_*i*_. However, we could assume another relationship between the independent variable and the dispersion index. Therefore, from now, we will consider the dispersion coefficient *δ* not to depend on *x*_*i*_, and then the domain of the parameters is *Θ*={*β*,*δ*}. Note that for the Poisson model *δ*=1 and the domain of the parameters is just *Θ*={*β*}.

Let *p*(*y*_*ij*_=*k*)=*p*(*k*|*μ*,*δ*) be the probability mass function of the model, parametrized in terms of its population mean and dispersion index. It is clear that *p*(*y*_*ij*_=*k*)=*p*(*k*|*f*(*x*_*i*_,*β*),*δ*)=*p*(*k*|*x*_*i*_,*Θ*), and then the likelihood function of the calibration data *D* becomes

2.2 $$L(D|\Theta )=\prod_{\begin{array}{c} i=1,\ldots ,n\\ j=1,\ldots ,n_{i} \end{array}}p(y_{ij}|x_{i},\Theta ).$$

According to the IAEA manual, the parameters are estimated by maximizing the likelihood function (2.2), obtaining $\hat{\Theta }=\{\hat{\beta },\hat{\delta }\}$. It is well known that for large data samples, the distribution of $\Theta \in R^{p+1}$ can be approximated by a multivariate Gaussian distribution $N_{p+1}(\hat{\Theta },{\hat{\Sigma }}_{\hat{\Theta }})$, where ${\hat{\Sigma }}_{\hat{\Theta }}$ is its estimated variance–covariance matrix, that is, the inverse of the estimated Fisher information matrix of the model. Note, however, that in the frequentist framework $\hat{\Theta }∼N_{p+1}(\Theta ,{\hat{\Sigma }}_{\hat{\Theta }})$. It is important to remark that the laboratory providing the outputs of the calibration curve, that is $\hat{\Theta }$ and ${\hat{\Sigma }}_{\hat{\Theta }}$, could be different from the one analysing the patient sample; even though for a consistent assay, the calibration curve should be constructed with the data provided by the same laboratory that will analyse the patient data to guarantee that the scoring criteria applied for the construction of the curve are the same as those applied for patient analysis.

From here, the distribution of the expected count of dicentrics and dispersion index for a given dose of *x*, (*μ*,*δ*)|*x* can be approximated by a bivariate normal distribution. This is a straightforward consequence of the multivariate delta method [19]

2.3 $$(\mu ,\delta )|x∼N_{2}((f(x,\hat{\beta }),\hat{\delta }),\nabla \cdot {\hat{\Sigma }}_{\hat{\Theta }}\cdot \nabla^{t}),$$

where ∇ denotes the derivative of (*f*(*x*,*β*),*δ*) at $\left(\hat{\beta },\hat{\delta }\right)$, that is,

$$\nabla =\left(\begin{array}{cccc} \frac{\partial f}{\partial \beta_{0}} & \cdots & \frac{\partial f}{\partial \beta_{p}} & 0\\ 0 & \cdots & 0 & 1 \end{array}\right).$$

Following these arguments, note that for the Poisson model the distribution of *μ*|*x* is approximated by a univariate normal distribution with expectation $f(x,\hat{\beta })$ and variance equal to $v(x,\hat{\beta })=\nabla \cdot {\hat{\Sigma }}_{\hat{\Theta }}\cdot \nabla^{t}$, where ∇ is now the gradient of *f*(*x*,*β*) at $\hat{\beta }$. The bivariate normal density in (2.3) will be denoted as *ϕ*(*μ*,*δ*|*x*) and *ϕ*(*μ*|*x*) will be the normal univariate density used for the Poisson model. In some situations, the use of a bivariate or univariate normal could be incompatible with the fact that *μ*>0, and in general *δ*>1. Then, some approximations have to be carried restricting the parameters' domain. For the univariate normal distribution, one solution is to replace it by a gamma density with the same mean and variance. It is well known that a larger gamma distribution shape parameter (i.e. the ratio of the square of the mean to the variance) implies a better normal approximation. As we will see in the next sections, the normal approximation can be used in a wide range of situations, and it also will be compared with the gamma approximation. For our purposes *μ*|*x* will be called the mean prior distribution, because it will act as a prior for the inverse regression estimation problem.

Consider the test (patient) data $\tilde{y}=\{{\tilde{y}}_{1},{\tilde{y}}_{2},\ldots ,{\tilde{y}}_{m}\}$, formed by *m* count data observations depending on an unknown regressor *x* that we aim to estimate. The likelihood function of the test data becomes

2.4 $$L(\tilde{y}|\mu ,\delta )=\prod_{i=1}^{m}p({\tilde{y}}_{i}|\mu ,\delta ).$$

Note that, because the knowledge of *μ* implies the knowledge of *x*, then we can write $L(\tilde{y}|\mu ,\delta )=L(\tilde{y}|\mu ,\delta ,x)$. Therefore, an application of Bayes' theorem shows the expression of the posterior density of the parameters given the test data

$$f(\mu ,\delta ,x|\tilde{y})=\frac{L(\tilde{y}|\mu ,\delta )p(\mu ,\delta ,x)}{\int L(\tilde{y}|\mu ,\delta )p(\mu ,\delta ,x)\;d\mu \;d\delta \;dx},$$

where p(*μ*,*δ*,*x*) is the joint prior density of *μ*, *δ* and *x*. But, p(*μ*,*δ*,*x*)=*ϕ*(*μ*,*δ*|*x*)p(*x*), where p(*x*) summarizes the prior information for *x*. This prior information can come from the characteristics of the radiation accident, such as the source and the duration of the exposure, etc.

Therefore, marginalizing over *μ* and *δ* we obtain the calibrative density of *x*, that it is the solution of the inverse regression problem

2.5 $$f(x|\tilde{y})\propto p(x)\int L(\tilde{y}|\mu ,\delta )\phi (\mu ,\delta |x)\;d\mu \;d\delta .$$

As shown in §3, this calibrative density can be exactly calculated for the Poisson model, solving completely the problem of the absorbed dose estimation in the most frequent situation.

However, for the two-parameter compound Poisson models the integral in (2.5) does not have a closed form, thus some approximations are required such as numerical integration or simulation methods. For this reason, the model will be simplified in §4.

## 3. The Poisson model

When data are Poisson distributed, the likelihood function of the test data has the form

$$L(\tilde{y}|\mu )\propto \prod_{i=1}^{m}p({\tilde{y}}_{i}|\mu )\propto e^{-m\mu }\mu^{\sum_{i=1}^{m}{\tilde{y}}_{i}}.$$

Because the sum of the observations is a sufficient statistic for the parameter of Poisson data, and the sum of independent Poisson random variables is also Poisson distributed, this likelihood function is equivalent to the probability function of one Poisson observation evaluated at *s*, that is,

$$L(\tilde{y}|\mu )\propto p(s|m\mu )\propto e^{-m\mu }{\left(m\mu \right)}^{s},$$

where $s=\sum_{i=1}^{m}{\tilde{y}}_{i}$. Therefore, the calibrative density (2.5) remains

3.1 $$f(x|\tilde{y})=p(x)q_{s}(x),$$

where

3.2 $$q_{s}(x)=\int_{-\infty }^{\infty }p(s|m\mu )\phi (\mu |x)\;d\mu .$$

Note that (3.2) represents the probability function of a mixed Poisson–normal distribution evaluated at *s*. Of course, strictly speaking, it is not possible to mix a Poisson with a normal distribution because the Poisson parameter always has to be positive. However, understanding this mixture as a purely formal operation, Kemp & Kemp [20] showed that this mixed Poisson distribution, provided the population mean of the mixing normal is greater than its variance, is just the Hermite distribution. Specifically, using Gurland's notation ([16,17]) we have the symbolic representation

$$\mathrm{Pois}(m\mu )\underset{\mu }{⋀}N(a,b^{2})∼\mathrm{Herm}\left(ma,1+\frac{mb^{2}}{a}\right).$$

This notation means that the *μ* parameter in the Poisson distribution (left part) is normally distributed (right part). This representation is valid only for *a*≥*mb*^2^.

Consequently, (3.2) is the probability that a Hermite random variable takes a value equal to *s*. Specifically, it can be directly shown that the probability (3.2) can be obtained from the Hermite probability recursion described in [21]

$$(r+1)q_{r+1}(x)=(mf(x,\hat{\beta })-m^{2}v(x,\hat{\beta }))q_{r}(x)+m^{2}v(x,\hat{\beta })q_{r-1}(x),$$

with $q_{0}(x)=\exp(-mf(x,\hat{\beta })+m^{2}v(x,\hat{\beta })/2)$ and defining *q*_−1_(*x*)=0, provided that $f(x,\hat{\beta })-mv(x,\hat{\beta })\geq 0$. This last inequality is achieved for most of the studied examples, for the range of interest of the absorbed dose *x*. In a hypothetical situation where this inequality was not achieved, that is $f(x,\hat{\beta })-mv(x,\hat{\beta })<0$, expression (3.2) mathematically does not make sense (the dispersion coefficient cannot be greater than 2) and it is therefore better to replace the mean prior normal density *ϕ*(*μ*|*x*) by a gamma density *Γ*(*μ*|*x*) with the same mean $f(x,\hat{\beta })$ and variance $v(x,\hat{\beta })$. Then, expression (3.2) would remain

3.3 $$q_{s}(x)=\int_{0}^{\infty }p(s|m\mu )\Gamma (\mu |x)\;d\mu .$$

Because mixing a Poisson with a gamma produces an NB distribution, it can be shown that *q*_*s*_(*x*) in (3.3) is the probability that an NB random variable, with mean $mf(x,\hat{\beta })$ and variance $m^{2}v(x,\hat{\beta })+mf(x,\hat{\beta })$, takes a value equal to *s*.

The method presented here for the Poisson model, using the gamma distribution as a mean prior, is exactly the same as the full Bayesian method of Groer & Pereira [9] for the simple case where *f*(*x*,*β*)=*βx*. However for other dose–response curves both methods differ. For this simple linear dose–response case, considering a uniform dose prior, direct calculations show that

$$f(x|\tilde{y})=\frac{m^{s+1}{\left(\sum n_{i}x_{i}\right)}^{\sum y_{i}}}{B(s+1,\sum y_{i}-1)}\frac{x^{s}}{{\left(mx+\sum n_{i}x_{i}\right)}^{s+\sum y_{i}}};$$

with mean, mode and variance of

$$\begin{array}{rl} M_{|\tilde{y}} & =\frac{s}{m}\frac{\sum n_{i}x_{i}}{\sum y_{i}},\\ E_{|\tilde{y}} & =\frac{\sum n_{i}x_{i}}{m}\frac{B(s+2,\sum y_{i}-2)}{B(s+1,\sum y_{i}-1)}\\ \mathrm{and}\;V_{|\tilde{y}} & =\frac{E_{|\tilde{y}}}{m}\cdot \left[\left(\sum n_{i}x_{i}-2\right)B\left(s+2,\sum y_{i}-2\right)+2B\left(s+3,\sum y_{i}-3\right)\right]; \end{array}$$

according to notation in §2, where B(⋅) denotes Euler's Beta function. The distribution function of this calibrative density can be expressed in terms of the hypergeometric function.

The following example illustrates how this methodology is applied to a real dataset.

### (a) Example: Cobalt-60 gamma rays irradiation

Here we consider data from an inter-laboratory comparison for the semi-automated dicentric assay undertaken as part of the Multibiodose project (a large-scale European biodosimetry project) [22]. This dataset (table 1) is based on blood samples from eight healthy donors which were irradiated *in vitro* with cobalt-60 gamma rays at a high-dose rate of $0.27\;\mathrm{Gy}\;\min^{-1}$ simulating acute whole body exposure. The data presented here were collated and analysed using the Metafer 4 automated analysis system (MetaSystems, Altlussheim, Germany) at a single participating laboratory, using the ‘BfS’ image analysis classifier (system settings—further information in Romm *et al.* [22]).

**Table 1.** Frequency distributions of the number of dicentrics after exposure to six doses of gamma rays, and the sample means, dispersion coefficients and *u* values for each distribution. Test data in italics.

	no. dicentrics							
dose (Gy)	0	1	2	3	4	$\bar{y}$	*d*	*u*
0.25	2185	8				0.004	0.997	−0.113
0.75	2550	44	1			0.018	1.026	0.952
1.00	2231	54	2			0.025	1.044	1.503
*1.50*	*1712*	*96*	*3*			*0.056*	*1.003*	*0.092*
2.50	1196	123	7	1		0.105	1.038	0.985
3.00	1070	320	41	6	1	0.295	1.012	0.334

The *u* figures shown in table 1 are the values of the *u*-test statistic of Rao & Chakravarti [23], which is a normalized sample dispersion index

$$u=(d-1)\sqrt{\frac{n-1}{2(1-1/z)}},$$

where $d=s_{y}^{2}/\bar{y}$ is the sample dispersion coefficient, *n* the sample size (number of cells) and $z=n\bar{y}$ the total number of count events (number of dicentrics). When *d* is close to 1 then the data follow an equidispersed distribution. If the value of the *u* statistic is higher (lower) than (-)2, the distribution can be considered over- (under-) dispersed. The *u*-test is suggested by the IAEA [3] and in fact it is equivalent to the classical Fisher dispersion test. According to the *u* values shown in table 1, equidispersion of the calibration data can be assumed, thus justifying the use of a Poisson regression model.

The 1.5 Gy row was removed from the calibration dataset to be used as test data. This means that the true dose is known and it is possible to compare it with the resulting calibrative density. Following notation in §3, *s*=102 and *m*=1811, i.e. 102 scored dicentrics in 1811 blood cells.

**Figure 1.**
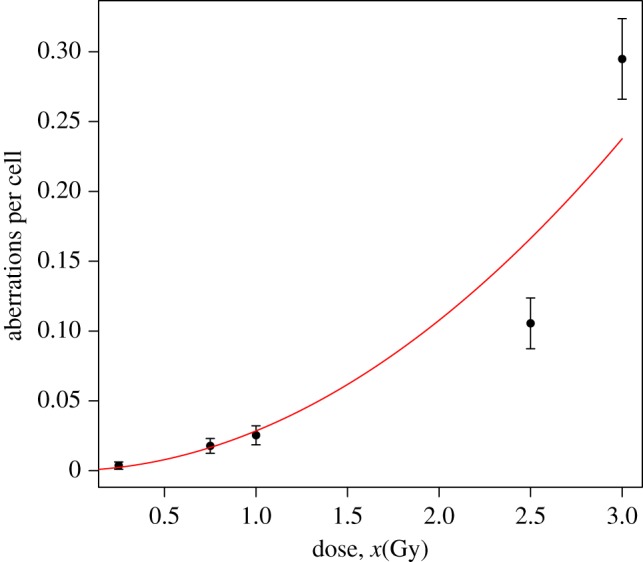
Observed means (dots), plus/minus twice their standard errors (error bars), and predicted means (solid line) of the number of dicentrics for Poisson fitting, based on the data in table 1, omitting the 1.5 Gy test data. (Online version in colour.)

In this example, for high-dose rate gamma-radiation exposure, an appropriate dose–response curve, i.e. the regression model, is a second degree polynomial without intercept [3], f(*x*,*β*)=*β*_2_*x*^2^+*β*_1_*x* (figure 1). In biodosimetry, this is called the linear-quadratic dose–response curve. The intercept has been removed because we assume that for a dose *x*=0 the expected number of dicentrics will be zero (for the 0 Gy sample there was only 1 dicentric in a total of 2592 blood cells). In general regression modelling, to analyse count data using a second degree polynomial mean response is not common, and a log-link mean response is the usual approach. However, in biodosimetry, the linear-quadratic dose–response curve has a biophysical interpretation [3] and is one of the most frequently employed in practice. Some problems could occur maximizing the likelihood function because *β*_1_ and *β*_2_ have to be necessarily positive. To ensure this, it is sometimes necessary to use numerical algorithms allowing constrains in the parameter domain.

 Table 2 shows the Bayesian information criterion (BIC) values for the four different response distributions treated in this work from the calibration data. These values support the use of the Poisson model. So for a Poisson response the maximum-likelihood parameter estimates and their estimated covariance matrix are the following:

— Fitted coefficients:
  $${\hat{\beta }}_{1}=3.126\times 10^{-3}\;\mathrm{and}\;{\hat{\beta }}_{2}=2.537\times 10^{-2}.$$
— Estimated covariance matrix:
  $${\hat{\Sigma }}_{\hat{\beta }}=\left(\begin{array}{cc} 7.205 & -3.438\\ -3.438 & 2.718 \end{array}\right)\times 10^{-6}.$$

**Table 2.** BIC values using a second degree polynomial dose–response curve without constant term for the different models.

model	NB	Hermite	NA	Poisson
BIC	4088.834	4085.594	4085.524	4079.639

As has been commented in §2, *μ*|*x* will follow a normal or a gamma distribution with mean $f(x,\hat{\beta })={\hat{\beta }}_{2}x^{2}+{\hat{\beta }}_{1}x$ and variance $v(x,\hat{\beta })=\nabla \cdot {\hat{\Sigma }}_{\hat{\beta }}\cdot \nabla^{t}$, where

$$\nabla =\left(\frac{\partial f}{\partial \beta_{1}},\frac{\partial f}{\partial \beta_{2}}\right)=(x,x^{2}),$$

and therefore $v(x,\hat{\beta })={\hat{\Sigma }}_{22}x^{4}+2{\hat{\Sigma }}_{21}x^{3}+{\hat{\Sigma }}_{11}x^{2}$.

According to (3.2) and (3.3), for a normal or a gamma mean prior, the predictive posterior distribution *q*_102_(*x*) represents the probability of a Hermite or NB random variable taking a value of 102 counts, both with same mean 45.939*x*^2^+5.661*x* and variance 8.913*x*^4^−22.553*x*^3^+69.571*x*^2^+5.661*x*.

Despite the real dose being known, firstly, a non-informative prior dose distribution is chosen in order to not take advantage of this fact, so p(*x*)∝1. Secondly, for our purposes of comparing results, we define an informative prior dose distribution assuming we do not know the real dose of the test data, but we observe a mean of 0.056 dicentrics per cell, then by comparison with table 1 it can reasonably be estimated that the dose is between 1 and 2.5 Gy. A simple informative prior could be a gamma whose mean is in the midpoint of this interval, i.e. 1.75, and whose standard deviation is in the halfway from the mean to cover this interval, i.e. 0.375. For a gamma distribution with this mean and standard deviation, the 95.67% of the values fall in the region of 1.75±2×0.375.

**Figure 2.**
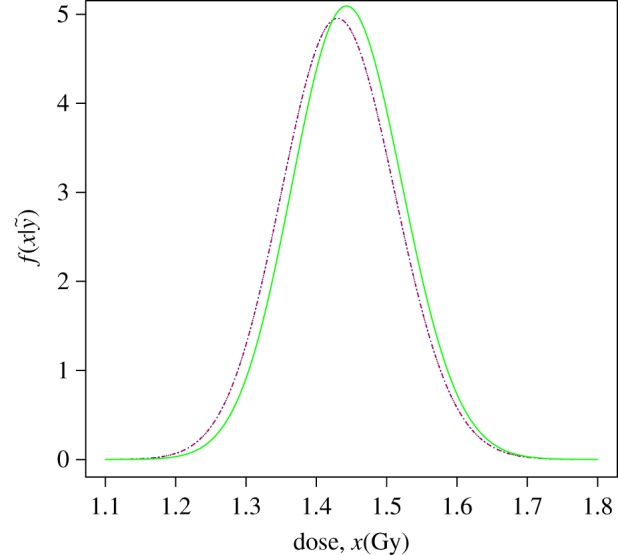
Calibrative densities of the 1.5 Gy test data calculated from a normal (blue/dotted line) and a gamma (red/dashed-dotted line) mean prior with non-informative prior dose distribution, and for a gamma mean prior with informative prior dose distribution (green/solid line). Red and blue curves are indistinguishable.

Figure 2 shows the plot of the three densities of the estimated dose for the data test. Note how these results incorporate the real dose (1.5 Gy) and show the similarities found using both mean priors. Note that the gamma mean prior is moderately more conservative.

To use the normal mean prior (3.2) for this calibration set, the following condition must be satisfied: $f(x,\hat{\beta })-mv(x,\hat{\beta })\geq 0$. It holds when *x*≤3.337 Gy, and this could also be used as prior information about the dose, that is, p(x)∼U(0,3.337). For the range of the likely doses studied, the minimum value of the shape parameter of the mean prior gamma is 328.616, so the gamma or normal mean priors are practically indistinguishable.

The statistics of the three calibrative densities calculated in this example are shown in table 3.

**Table 3.** Statistics summary of the calibrative densities for a normal (*a*) and a gamma (*b*) mean prior with non-informative prior dose distribution, and for a gamma mean prior with informative prior dose distribution (*c*).

model	mode	expected	s.d.	95% CI
(*a*)	1.430	1.432	0.081	(1.277, 1.594)
(*b*)	1.430	1.432	0.081	(1.277, 1.593)
(*c*)	1.443	1.445	0.078	(1.294, 1.602)

### (b) Example: analysis of doses in thyroid cancer patients

This example illustrates how our methodology can be applied having only the fitted parameters of the dose–response curve, without knowing the calibration points. Serna *et al.* [24] studied chromosomal damage in lymphocytes of thyroid cancer patients after radioiodine treatment. The authors did a micronuclei assay in binucleated cells of blood samples from 25 patients 3 days after Iodine-131 (3.7 GBq) exposure.

The *in vitro* calibration curve was fitted by a linear-quadratic model with intercept, f(*x*,*β*)=*Gβ*_2_*x*^2^+*β*_1_*x*+*β*_0_ according to Poisson's law, and the estimate of *β*_0_ was not taken into account, because the authors in [24] argued that the intercept could change for each patient. Constant *G* is the Lea–Catcheside generalized dose-protraction factor, which modifies the quadratic term according to the temporal pattern of exposure, being *G*=1 for the **in vitro** assay. The authors calculated the following parameter estimates (${\hat{\beta }}_{i}\pm \mathrm{SE}({\hat{\beta }}_{i}$))

$${\hat{\beta }}_{1}=(13.6\pm 5.5)\times 10^{-3},\;{\hat{\beta }}_{2}=(3.7\pm 1.6)\times 10^{-2},\;\rho =-0.89,$$

where *ρ* is the correlation coefficient for ${\hat{\beta }}_{1}$ and ${\hat{\beta }}_{2}$. The patients were subjected to ablative radioiodine treatments for post-surgical thyroid remnants. Consequently, they had a prolonged exposure lasting several days and which means, the temporal pattern of exposure was different than that of the **in vitro** assay. Taking into account the exposure profile of the Iodine-131 treatment, the authors in [24] found the factor G to be close to 0.1.

Then *β*_0_, the background for each patient, can be estimated counting the micronuclei of the patient from a blood sample taken before the treatment, information provided in [24]. This leads to the fitted regression model $f(x,\hat{\beta })=G{\hat{\beta }}_{2}x^{2}+{\hat{\beta }}_{1}x+{\hat{\beta }}_{0}$ with a covariance matrix that incorporates the variance of ${\hat{\beta }}_{0}$ without correlation with ${\hat{\beta }}_{1}$ and ${\hat{\beta }}_{2}$.

To illustrate our techniques we are going to estimate the absorbed dose for Patient 1, but the same can be done for the others. Patient 1 presented 487 normal cells and 13 cells with just one micronucleus each. Before the treatment five micronuclei where found in 500 blood cells, thus ${\hat{\beta }}_{0}=(10\pm 4.450)\times 10^{-3}$. The u-statistic of the test data is −0.395, so this is compatible with the Poisson model.

Therefore, *μ*|*x* will be considered to follow a distribution with mean $f(x,\hat{\beta })=G{\hat{\beta }}_{2}x^{2}+{\hat{\beta }}_{1}x+{\hat{\beta }}_{0}$ and variance $v(x,\hat{\beta })=\nabla \cdot {\hat{\Sigma }}_{\hat{\beta }}\cdot \nabla^{t}$, where

$$\nabla =\left(\frac{\partial f}{\partial \beta_{0}},\frac{\partial f}{\partial \beta_{1}},\frac{\partial f}{\partial \beta_{2}}\right)=(1,x,Gx^{2}).$$

The condition $f(x,\hat{\beta })-mv(x,\hat{\beta })\geq 0$ is held when *x*≤0.880 Gy. This range of doses is very small for our purposes and consequently a gamma mean prior is preferred instead of a normal.

According to (3.3), for a gamma mean prior, the predictive posterior distribution q_13_(*x*) represents the probability of an NB random variable taking a value of 13 counts, with mean 0.185*x*^2^+6.8*x*+5 and variance 0.006*x*^4^−0.399*x*^3^+7.987*x*^2^+6.8*x*+9.95.

Three calibrative densities have been calculated applying two different proper uniform prior dose distributions, both using information given in [24]. An administered radioiodine activity that produces a blood dose less than 2 Gy is considered safe, so we could take a uniform dose prior distribution from 0 to 2, assuming that doctors use prudent doses. On the other hand, the calibration curve was calculated up to a dose of 4.5 Gy, so another uniform dose prior distribution could be from 0 to 4.5. An improper uniform prior dose distribution from 0 to +∞ is also applied.

**Figure 3.**
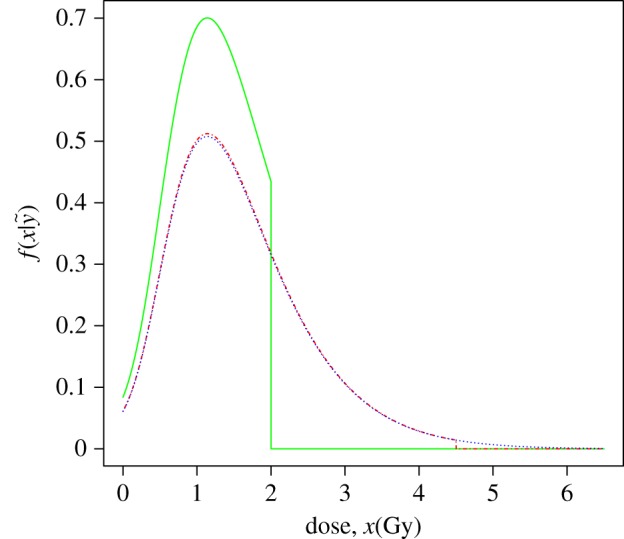
Calibrative densities of [24] Patient 1 test data calculated from a gamma mean prior density, with a U(0,2) (green/solid line), a U(0,4.5) (red/dashed-dotted line) prior dose distribution and a improper U(0,+∞) (blue/dotted line) prior dose distribution.

 Figure 3 shows the plot of the three densities of the estimated dose for the data test. Their statistics are indicated in table 4. These results agree with those displayed in [24], where the dose estimate for Patient 1 was 1.14 Gy.

**Table 4.** Statistics summary of the calibrative densities for two proper and one improper uniform dose priors.

prior dose distribution	mode	expected	s.d.	95% CI
U(0,2)	1.140	1.141	0.481	(0.203,1.945)
U(0,4.5)	1.140	1.561	0.858	(0.203,3.615)
U(0,+∞)	1.140	1.593	0.921	(0.253,3.829)

## 4. The simplified compound Poisson calibration model

We now consider a dataset that follows a compound Poisson distribution. The likelihood function of the test data has been previously described in (2.4), and the calculation of the calibrative density (2.5) requires to use numerical integration or Monte Carlo methods. However, the model can be simplified by replacing *δ* in $L(\tilde{y}|\mu ,\delta )$ with the MLE $\hat{\delta }$ obtained from the calibration data. The performance of this simplification is analysed and compared in the example §3*a*. Then the likelihood function $L(\tilde{y}|\mu ,\hat{\delta })$, which we prefer to denote as $L(\tilde{y},\hat{\delta }|\mu )$, is equivalent to the probability function of the sum of the observations, that is the probability function of a compound Poisson observation,

$$L(\tilde{y},\hat{\delta }|\mu )\propto p(s,\hat{\delta }|m\mu ),$$

where $s=\sum_{i=1}^{m}{\tilde{y}}_{i}$. Then, the calibrative density is as described in (3.1) with

4.1 $$q_{s}(x)=\int_{-\infty }^{\infty }p(s,\hat{\delta }|m\mu )\phi (\mu |x)\;d\mu$$

if the mean prior is a normal density, or

4.2 $$q_{s}(x)=\int_{0}^{\infty }p(s,\hat{\delta }|m\mu )\Gamma (\mu |x)\;d\mu$$

when the mean prior is gamma distributed. Expressions (4.1) and (4.2) correspond to the probability function of mixed compound Poisson random variables, where the mixing density is respectively normal or gamma, evaluated at *s*. The operations of compounding and mixing are interchangeable for these models [16,17], e.g. mixing an NA with a normal result in the following:

4.3 $$\begin{array}{rl} & \mathrm{NA}(m\mu ,\hat{\delta })\underset{\mu }{⋀}N(f(x,\hat{\beta }),v(x,\hat{\beta }))\\ & \;=\mathrm{Pois}\left(\frac{m\mu }{\hat{\delta }-1}\right)⋁\mathrm{Pois}(\hat{\delta }-1)\underset{\mu }{⋀}N(f(x,\hat{\beta }),v(x,\hat{\beta }))\\ & \;=\mathrm{Pois}\left(\frac{m\mu }{\hat{\delta }-1}\right)\underset{\mu }{⋀}N(f(x,\hat{\beta }),v(x,\hat{\beta }))⋁\mathrm{Pois}(\hat{\delta }-1)\\ & \;=\mathrm{Herm}\left(\frac{mf(x,\hat{\beta })}{\hat{\delta }-1},1+\frac{mv(x,\hat{\beta })}{\left(\hat{\delta }-1)f(x,\hat{\beta }\right)}\right)⋁\mathrm{Pois}(\hat{\delta }-1). \end{array}$$

This is providing that (4.1) and (4.2) are, respectively, the probability functions of compound Hermite and compound NB random variables. Therefore, according to the different choices of the compound Poisson distribution we obtain the following compound distributions for *q*_*s*_(*x*):

4.4 $$\begin{array}{rl} & \mathrm{NA}:\;F\left(\frac{mf(x,\hat{\beta })}{\hat{\delta }-1},1+\frac{mv(x,\hat{\beta })}{\left(\hat{\delta }-1)f(x,\hat{\beta }\right)}\right)⋁\mathrm{Pois}(\hat{\delta }-1),\\ & \mathrm{NB}:\;F\left(\frac{mf(x,\hat{\beta })\log(\hat{\delta })}{\hat{\delta }-1},1+\frac{mv(x,\hat{\beta })\log(\hat{\delta })}{\left(\hat{\delta }-1)f(x,\hat{\beta }\right)}\right)⋁\log\left(\frac{\hat{\delta }-1}{\hat{\delta }}\right)\\ \text{and}\; & \mathrm{Hermite}:\;F\left(\frac{mf(x,\hat{\beta })}{2(\hat{\delta }-1)},1+\frac{mv(x,\hat{\beta })}{2(\hat{\delta }-1)f(x,\hat{\beta })}\right)⋁\mathrm{Bin}(2,\hat{\delta }-1). \end{array}\}$$

Here $F(\mu_{F},\delta_{F})$ indicates a Hermite or an NB distribution, according to (4.1) or (4.2), parametrized by its population mean and dispersion index. When F is the Hermite distribution, these representations make sense only when $f(x,\hat{\beta })(\hat{\delta }-1)\geq mv(x,\hat{\beta })$ for the NA, $f(x,\hat{\beta })(\hat{\delta }-1)\geq mv(x,\hat{\beta })\log(\hat{\delta })$ for the NB and $2f(x,\hat{\beta })(\hat{\delta }-1)\geq mv(x,\hat{\beta })$ for the Hermite.

Compound NB distributions have been studied and applied in several publications. Properties, characterizations and references can be found in [16]. Compound Hermite distributions are less common, so far there is one recent publication [25] that studies the continuous compound Hermite gamma distribution.

When $F(\mu_{F},\delta_{F})$ is NB, the probabilities of the associated compound distributions can be calculated using the Panjer recursion formula [26]. This formula is based on the fact that the probabilities *p*_*n*_=*P*(*X*=*n*) of a random variable *X* distributed as a NB$\left(\mu_{F},\delta_{F}\right)$ satisfy a first-order recurrence relation *p*_*n*_=*p*_*n*−1_(*a*+*b*/*n*), where $a=(\delta_{F}-1)/\delta_{F}$ and $b=(\mu_{F}-\delta_{F}+1)/\delta_{F}$. Then, if the probabilities of the generalizing distribution are denoted as *f*_*k*_, the probabilities *q*_*i*_ of the corresponding NB compound distribution satisfy the recursion [26]

4.5 $$q_{0}=\frac{p_{0}}{{\left(1-f_{0}a\right)}^{1+b/a}}\;\mathrm{and}\;q_{i}=\sum_{j=1}^{i}\left(a+\frac{bj}{i}\right)f_{j}q_{i-j},\;i\geq 1.$$

Expression (4.5) can be efficiently used to calculate (4.2). The values of *a* and *b* will be taken according to the chosen distribution of the observations, using the corresponding expression of $\mu_{F}$ and $\delta_{F}$ of the NB (F) indicated in (4.4). In the next section we will give an example of application.

When F is Hermite, the probabilities of a Hermite compound distribution cannot be calculated using the Panjer recursion formula because the probabilities of the Hermite do not follow a linear recursion. To calculate the probabilities in this case we state and prove (in appendix A) the following proposition:

**Proposition 4.1**

*Let* *q*_*n*_, *n*=0,1,2… *be the probabilities of a compound Hermite distribution of the form Herm* $(\mu_{h},\delta_{h})⋁P,$ *where* P *is a count distribution with probabilities* *f*_*k*_, *k*=0,1,2…. *We define* $r_{j}=\sum_{i=0}^{j}f_{i}f_{j-i},\;j=0,1,2\ldots ,$ *then*

4.6 $$q_{n}=\frac{\mu_{h}}{n}\sum_{i=0}^{n-1}(n-i)q_{i}\left\{(2-\delta_{h})f_{n-i}+\frac{\left(\delta_{h}-1\right)}{2}r_{n-i}\right\},$$

*and* $q_{0}=\exp(\mu_{h}((2-\delta_{h})(f_{0}-1)+(\delta_{h}-1)(f_{0}^{2}-1)/2))$.

It is important to remark that, to calculate *q*_*s*_(*x*) in (4.1) and (4.2), a computationally intensive direct numerical integration can be done instead to use the Panjer recursion or proposition 4.1. To this end, it would be enough to obtain numerically the probabilities which are available for a more wide range of models than those studied in this paper.

The use of (4.6) will be illustrated with a real data analysis in the next section.

### (a) Example: high linear energy transfer exposure

Puig & Valero [18] studied the fitting of an experiment of 11 samples of peripheral blood exposed to different doses of *γ*-rays (table 5), where the dose rate was $0.93\;\mathrm{cGy}\min^{-1}$. For each sample, approximately 5000 binucleated cells were inspected, and the numbers of micronuclei were counted.

**Table 5.** Frequency distributions of the number of micronuclei after exposure to 11 doses of gamma rays, and the sample means, dispersion coefficients and *u* values for each distribution. Test data in italics.

	no. micronuclei										
dose (Gy)	0	1	2	3	4	5	6	7	$\bar{y}$	*d*	*u*
0.00	4887	106	5	2					0.024	1.156	7.839
*0.10*	*4773*	*206*	*19*	*2*					*0.050*	*1.150*	*7.526*
0.25	4261	324	41	12	2				0.090	1.306	15.306
0.50	4536	364	76	17	7				0.119	1.449	22.484
0.75	4383	512	85	18	2				0.149	1.257	12.876
1.00	4225	636	115	19	5				0.189	1.240	12.009
1.50	4018	805	139	26	9	1	2		0.243	1.270	13.495
2.00	3499	1194	238	45	13	10	1		0.383	1.209	10.471
2.50	3171	1313	393	94	24	3	2		0.501	1.201	10.077
3.00	2582	1575	598	190	44	9	2	6	0.722	1.206	10.307
4.00	1974	1674	869	342	102	26	13	2	1.013	1.172	8.628

The *u* values shown in table 5 confirm the overdispersion, thus Poisson regression is not adequate.

Similar to the example analysed in §3*a* the 0.1 Gy data will be removed to be used as test data. This distribution has a total of 250 micronuclei in a total of 5000 cells so *s*=250 and *m*=5000.

**Figure 4.**
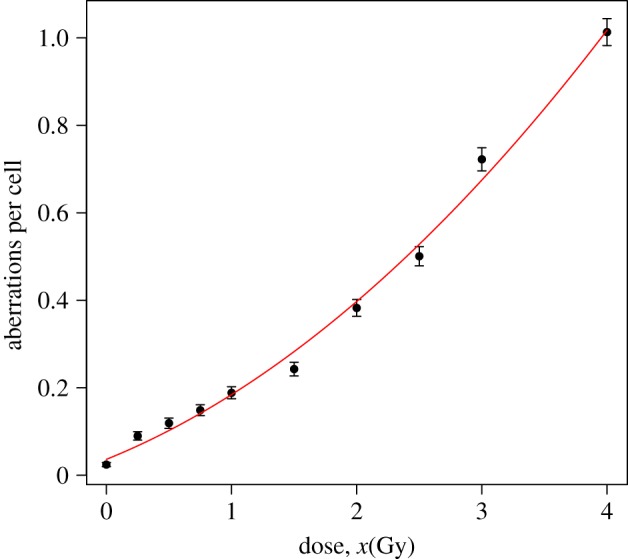
Observed means (dots), plus/minus twice their standard errors (error bars), and predicted means (solid line) of the number of micronuclei for NB fitting, based on the data in table 5, omitting the 0.1 Gy test data. (Online version in colour.)

The appropriate dose–response curve, i.e. the regression model, is again a linear-quadratic model with intercept, *f*(*x*,*β*)=*β*_2_*x*^2^+*β*_1_*x*+*β*_0_ (figure 4). Table 6 shows the BIC values for the four different models studied in this work. Note how these values support the use of the NB model.

**Table 6.** BIC values using a second degree polynomial dose–response curve for the different models.

model	Poisson	Hermite	NA	NB
BIC	67360.01	66537.46	66467.85	66437.93

Using the NB model, the maximum-likelihood estimation provides the following results:

— Fitted coefficients:
  $${\hat{\beta }}_{0}=3.639\times 10^{-2},\;{\hat{\beta }}_{1}=1.156\times 10^{-1},\;{\hat{\beta }}_{2}=3.241\times 10^{-2},\;\hat{\delta }=1.231.$$
— Estimated covariance matrix:
  $${\hat{\Sigma }}_{\hat{\Theta }}=\left(\begin{array}{cccc} 73.749 & -115.908 & 29.210 & 13.976\\ -115.908 & 373.338 & -110.398 & 36.919\\ 29.210 & -110.398 & 38.102 & -3.625\\ 13.976 & 36.919 & -3.625 & 1133.825 \end{array}\right)\times 10^{-7}.$$

Then, the prior densities are:

— Complete Model: According to (2.3), (*μ*,*δ*)|*x* follows a bivariate normal distribution with mean $\left({\hat{\beta }}_{2}x^{2}+{\hat{\beta }}_{1}x+{\hat{\beta }}_{0},\hat{\delta }\right)$ and variance–covariance $\nabla \cdot {\hat{\Sigma }}_{\hat{\Theta }}\cdot \nabla^{t}$, where
  $$\nabla =\left(\begin{array}{cccc} \frac{\partial f}{\partial \beta_{0}} & \frac{\partial f}{\partial \beta_{1}} & \frac{\partial f}{\partial \beta_{2}} & 0\\ 0 & 0 & 0 & 1 \end{array}\right)=\left(\begin{array}{cccc} 1 & x & x^{2} & 0\\ 0 & 0 & 0 & 1 \end{array}\right),$$
  so the variance-covariance is
  $$\left(\begin{array}{cc} {\hat{\Sigma }}_{33}x^{4}+2{\hat{\Sigma }}_{32}x^{3}+2{\hat{\Sigma }}_{31}x^{2}+{\hat{\Sigma }}_{22}x^{2}+2{\hat{\Sigma }}_{21}x+{\hat{\Sigma }}_{11} & {\hat{\Sigma }}_{43}x^{2}+{\hat{\Sigma }}_{42}x+{\hat{\Sigma }}_{41}\\ {\hat{\Sigma }}_{43}x^{2}+{\hat{\Sigma }}_{42}x+{\hat{\Sigma }}_{41} & {\hat{\Sigma }}_{44} \end{array}\right).$$
  For this example, the calibrative density (2.5) is calculated via numerical integration in order to be compared with those calculated using the simplified models.
— Simplified Models: According to the arguments given in §4, *μ*|*x* follows a gamma or a normal distribution with mean $f(x,\hat{\beta })={\hat{\beta }}_{2}x^{2}+{\hat{\beta }}_{1}x+{\hat{\beta }}_{0}$ and variance $v(x,\hat{\beta })=\nabla \cdot {\hat{\Sigma }}_{\beta }\cdot \nabla^{t}$, where
  $$\nabla =\left(\frac{\partial f}{\partial \beta_{0}},\frac{\partial f}{\partial \beta_{1}},\frac{\partial f}{\partial \beta_{2}}\right)=(1,x,x^{2}),$$
  so the variance is ${\hat{\Sigma }}_{33}x^{4}+2{\hat{\Sigma }}_{32}x^{3}+2{\hat{\Sigma }}_{31}x^{2}+{\hat{\Sigma }}_{22}x^{2}+2{\hat{\Sigma }}_{21}x+{\hat{\Sigma }}_{11}$. According to (4.4), for a normal or a gamma mean prior, the predictive posterior distribution *q*_250_(*x*) represents respectively the probability of a compound Hermite- or compound NB-Logarithmic random variable taking a value of 250 counts, both with same $f(x,\hat{\beta })=0.032x^{2}+0.116x+0.036$, $v(x,\hat{\beta })=3.81\times 10^{-6}x^{4}+1.525\times 10^{-5}x^{3}+5.842\times 10^{-6}x^{2}-2.318\times 10^{-5}x+7.375\times 10^{-6}$ and $\hat{\delta }=1.231$.

**Figure 5.**
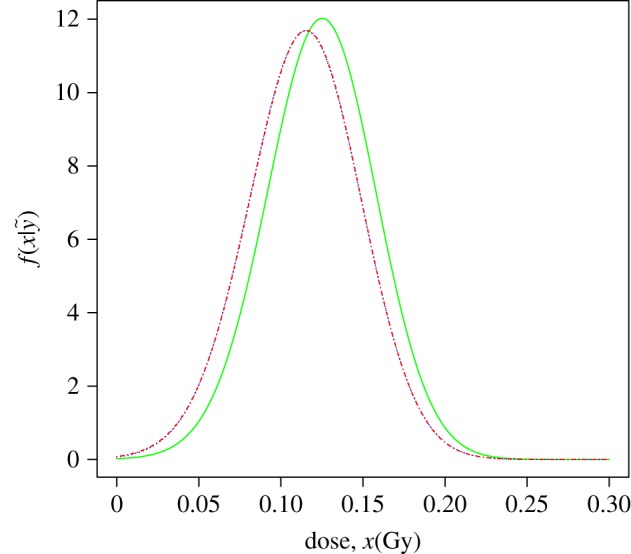
Calibrative densities of the 0.1 Gy test data using the complete model (2.5) (green/solid line), and the simplified ones with a normal (blue/dotted line) and a gamma (red/dashed-dotted line) mean prior density; all with a uniform prior dose distribution. Blue and red curves are indistinguishable.

To use the normal mean prior (4.1) in this calibration set for NB responses, there is a condition to be satisfied: $f(x,\hat{\beta })(\hat{\delta }-1)-mv(x,\hat{\beta })\log(\hat{\delta })\geq 0$. It is satisfied when *x*≤4.294 Gy. In this example, this is not a problem and it could be used as prior information about the dose, that is p(x)∼U(0,4.294). For the range of the likely doses studied, the minimum value of the shape parameter of the mean prior gamma is 179.605, and consequently both gamma and normal mean priors are almost indistinguishable (red and blue curves in figure 5).

Figure 5 shows the plot of the three densities (one from the complete model and two from the simplified ones) of the estimated dose for the data test. Note that both calibrative densities from the simplified models are practically the same. The statistics of these densities are shown in table 7. These results incorporate the real dose (0.1 Gy) and also show their similarities, chiefly between the simplified models.

**Table 7.** Statistics summary of the calibrative densities for the complete model, and the simplified models using a gamma and a normal mean prior with a uniform prior dose distribution.

model	complete	S. Norm. p.	S. Gam. p.
mode	0.125	0.115	0.115
expected	0.124	0.114	0.114
s.d.	0.033	0.034	0.034
95% CILB	0.059	0.047	0.047
95% CIUB	0.190	0.182	0.181

## 5. Conclusion

In this paper, we have presented several Bayesian-type methods for count data inverse regression, showing its application in the field of cytogenetic dosimetry. First, in §2 we defined our methodology for inverse regression, where responses are either Poisson or two-parameter compound Poisson. We have assumed that the dispersion index is constant along the different doses. This methodology leads to a bivariate normal prior density when the responses follow a two-parameter compound Poisson distribution, and an univariate normal or gamma mean prior density when the responses follow a Poisson distribution. To use our methodology, only the estimates of the parameters and covariance matrix of the dose–response curve are required. This information is available from the standard frequentist analysis suggested by the IAEA manual, with many examples published by other researchers or laboratories. Therefore, our method is not a full Bayesian approach because the dose–response curve is estimated using frequentist analysis. MCMC methods could be used if the models were more complex or the prior densities more complicated. They might also be used for model averaging, since one might aim to avoid choosing one of the presented four models, preferring to use a weighted amalgam of them.

The Poisson model is developed in §3, leading to a closed form of the calibrative density. Two examples of dose estimation based on the dicentric assay are reported.

In §4, we treated two-parameter compound Poisson models, simplifying them to get the calibrative densities into a closed form. For this purpose, we have presented a method which involves calculating the probabilities of compound NB distributions, using Panjer's recursion [26], and compound Hermite distributions, using a recursion relation described in proposition 4.1. Another example of dose estimation is shown, based on data obtained with the micronucleus assay. We have assumed a constant dispersion coefficient, but our methods could be also extended to dose-dependent dispersion models of the form *δ*_*ij*_=*g*(*x*_*i*_,*γ*), $\gamma \in R^{q}$.

The illustrative examples show applications using the most frequent calibrative curves, that are second-order polynomials (the linear-quadratic model). However, other response functions can be directly analysed using the same methodology. It should be noted that the approaches presented here may also prove useful in areas other than biological dosimetry.

## Supplementary Material

Example 3 (a)

## Supplementary Material

Example 3 (a) model (a)

## Supplementary Material

Example 3 (b)

## Supplementary Material

Example 4 (a)

## Appendix A. Proof of proposition 4.1

First of all, let us recall some topics related to the probability-generating function (pgf). Given a count random variable *X*, its pgf *Φ*_*X*_(*s*) is defined as

$$\Phi_{X}(s)=\sum_{k=0}^{\infty }p_{k}s^{k},$$

where the coefficients of this power series are the probabilities *p*_*k*_=*P*(*X*=*k*) and consequently the derivatives at *s*=0 divided by *k*! provide the probability mass function of *X*. The pgf of a compound probability distribution described in (2.1) is

A 1 $$\Phi_{X}(s)=\Phi_{N}(\Phi_{\xi }(s)),$$

where *Φ*_*N*_(*s*) is the pgf of *N* and *Φ*_*ξ*_(*s*) is the common pgf of the *ξ*_*i*_ [16].

One property of pgf's is that the sum of independent random variables is a random variable whose pgf is the product of the pgf's of the summed variables; e.g. given *X* and *Y* independent random variables with pgf's *Φ*_*X*_(*s*) and *Φ*_*Y*_(*s*) respectively, the pgf of *X*+*Y* results

A 2 $$\Phi_{X+Y}(s)=\Phi_{X}(s)\Phi_{Y}(s).$$

According to Kemp & Kemp [20] the pgf of a random variable *X* Hermite distributed with mean *μ*_*h*_ and dispersion coefficient *δ*_*h*_ is

A 3 $$\Phi_{X}(s)=e^{\mu_{h}\{(2-\delta_{h})(s-1)+(\delta_{h}-1)(s^{2}-1)/2\}},$$

therefore, according to (A 1), the pgf of a Herm$(\mu_{h},\delta_{h})⋁P$ distribution, being *ψ*(*s*) the pgf of P, is

A 4 $$\phi (s)=e^{\mu_{h}\{(2-\delta_{h})(\psi (s)-1)+(\delta_{h}-1)(\psi^{2}(s)-1)/2\}},$$

thus the probability in 0 is

$$q_{0}=\phi (0)=e^{\mu_{h}\{(2-\delta_{h})(f_{0}-1)+(\delta_{h}-1)(f_{0}^{2}-1)/2\}}.$$

Note that *ψ*^2^(*s*) is the pgf of a sum of two independent identically distributed random variables having both a pgf equal to *ψ*, so

$$\varphi (s)=\psi^{2}(s)=\sum_{n=0}^{\infty }r_{n}s^{n},\;r_{n}=\sum_{i=0}^{n}f_{i}f_{n-i}.$$

The derivative of *ϕ* is

$$\phi^{\prime }(s)=\left[\mu_{h}\left\{(2-\delta_{h})(\psi^{\prime }(s)-1)+\frac{\left(\delta_{h}-1)(\varphi^{\prime }(s)-1\right)}{2}\right\}\right]\phi (s),$$

therefore,

$$\begin{array}{rl} \sum_{n=1}^{\infty }nq_{n}s^{n-1} & =\mu_{h}\left\{(2-\delta_{h})\sum_{n=1}^{\infty }nf_{n}s^{n-1}+\frac{\left(\delta_{h}-1\right)}{2}\sum_{n=1}^{\infty }nr_{n}s^{n-1}\right\}\sum_{n=0}^{\infty }q_{n}s^{n}\\ & =\mu_{h}\sum_{n=1}^{\infty }n\left\{(2-\delta_{h})f_{n}+\frac{\left(\delta_{h}-1\right)}{2}r_{n}\right\}s^{n-1}\sum_{n=0}^{\infty }q_{n}s^{n}, \end{array}$$

matching the coefficients with same degree in *s* in both sides leads to

$$q_{n}=\frac{\mu_{h}}{n}\sum_{i=0}^{n-1}(n-i)q_{i}\left\{(2-\delta_{h})f_{n-i}+\frac{\left(\delta_{h}-1\right)}{2}r_{n-i}\right\},\;n\geq 1,$$

and this finishes the proof.
